# Distinct Motor Strategies Underlying Split-Belt Adaptation in Human Walking and Running

**DOI:** 10.1371/journal.pone.0121951

**Published:** 2015-03-16

**Authors:** Tetsuya Ogawa, Noritaka Kawashima, Hiroki Obata, Kazuyuki Kanosue, Kimitaka Nakazawa

**Affiliations:** 1 Faculty of Sport Sciences, Waseda University, 2-579-15 Mikajima, Tokorozawa, Saitama, Japan; 2 Japan Society for the Promotion of Science, 5-3-1 Kojimachi, Chiyoda, Tokyo, Japan; 3 Department of Rehabilitation for the Movement Functions, Research Institute, National Rehabilitation Center for Persons with Disabilities, 4-1, Namiki, Tokorozawa, Saitama, Japan; 4 Graduate School of Arts and Sciences, The University of Tokyo, 3-8-1, Komaba, Meguro, Tokyo, Japan; University of Alberta, CANADA

## Abstract

The aim of the present study was to elucidate the adaptive and de-adaptive nature of human running on a split-belt treadmill. The degree of adaptation and de-adaptation was compared with those in walking by calculating the antero-posterior component of the ground reaction force (GRF). Adaptation to walking and running on a split-belt resulted in a prominent asymmetry in the movement pattern upon return to the normal belt condition, while the two components of the GRF showed different behaviors depending on the gaits. The anterior braking component showed prominent adaptive and de-adaptive behaviors in both gaits. The posterior propulsive component, on the other hand, exhibited such behavior only in running, while that in walking showed only short-term aftereffect (lasting less than 10 seconds) accompanied by largely reactive responses. These results demonstrate a possible difference in motor strategies (that is, the use of reactive feedback and adaptive feedforward control) by the central nervous system (CNS) for split-belt locomotor adaptation between walking and running. The present results provide basic knowledge on neural control of human walking and running as well as possible strategies for gait training in athletic and rehabilitation scenes.

## Introduction

Despite their highly stereotyped motor patterns derived from specialized functional networks in the central nervous system (CNS), human locomotion is regulated flexibly to meet changing tasks and environmental demands. It has been well documented that both rapid reactive and slower adaptive modifications of movement patterns take place. For example, when walking on a slippery surface without any past experience, we initially have to react to overcome unpredictable perturbations to avoid a fall. Through repeated practice, however, an appropriate way of walking is adapted on the basis of the past experiences. In the modifications of the movement patterns, the former (reaction) takes place by utilizing peripheral feedback while the latter (adaptation) reflects predictive feedforward control of the cerebellum (For review, see [1,2]).

As a useful model for elucidating both the reactive and adaptive nature of human locomotion, split-treadmill walking has been studied extensively over the last decade [3–15]. Exposure to a split-belt (asymmetry in velocity of two belts) initially results in immediate reactive responses and subsequently adaptive changes lasting over minutes. These aspects have been investigated by addressing both spatiotemporal [12] and kinetic gait parameters [10,11]. In the spatiotemporal parameters, for example, reactive responses are expressed as transient changes in modified stride length (reflecting the anterior-posterior distance traveled by the foot during the stance phase) and stance time [12]. Adaptive behaviors are most evident in step time, double support time, and relative timing of the two leg movements that in turn result in an emergence of aftereffect (asymmetrical movements stored previously through walking on the split-belt) upon return to the symmetrical belt condition [12]. Kinetically, the anterior braking component of the ground reaction force (GRF) shows clear adaptive and de-adaptive aspects while others such as the vertical, mediolateral, and posterior propulsive components show only transient reactive changes [11].

Along with serving as a useful model to reveal the nature of locomotor adaptability, use of the split-belt treadmill adaptation paradigm provides information on task- or context-specific functional networks underlying different gaits [3,10,15]. It can also lead to possible strategies for gait rehabilitation programs for patients with particular CNS lesions [4,13].

Provided the results in the previous studies, a potential issue underlying gait adaptation studies in the future may be the lack of information on other locomotor tasks, since the results demonstrated thus far are focused only on walking. As a representative example of further study, we addressed the characteristics of locomotor adaptation in running, which is another major form of locomotion in humans. While there is a similarity with respect to the repetition of flexion and extension movements in the joints of two multisegmental limbs, it is nevertheless held that walking and running are recognized as different in terms of the underlying neural control mechanisms [10,16,17]. It is then of particular interest that how adaptations (if any) take place (either similarly or differently in comparison to those in walking). Following this logic, the purpose of the present study was to compare the characteristics of locomotor adaptation in human walking and running on a split-belt treadmill. On the basis of our preceding results focusing on adaptive and de-adaptive behavior of walking [11], we specifically focused on the anterior braking and posterior propulsive components of the GRF.

## Methods

### Subjects

Nine male subjects (mean ± SD age, 25.7 ± 3.1 years old; height, 172.9 ± 4.9 cm; weight, 63.2 ± 7.3 kg) with no known history of neurological and orthopedic disorders volunteered for the study. Prior to participation, they gave written informed consent. The study was approved by the local ethics committee of the National Rehabilitation Center of Persons with Disabilities, Japan, and was conducted according to the Declaration of Helsinki.

### Experimental protocols

In the experiment, subjects walked and ran on a split-belt treadmill (Bertec, Columbus, OH, USA) in which two belts (one underneath each foot) can be operated at separate velocities. The treadmill was operated in either “tied” (two belts at the same velocity) or “split” (at different velocities) [12] modes depending on the time period. The fast moving side and the slow moving side during the adaptation period are referred to as the “fast side” and “slow side”, respectively.

**Fig 1:**
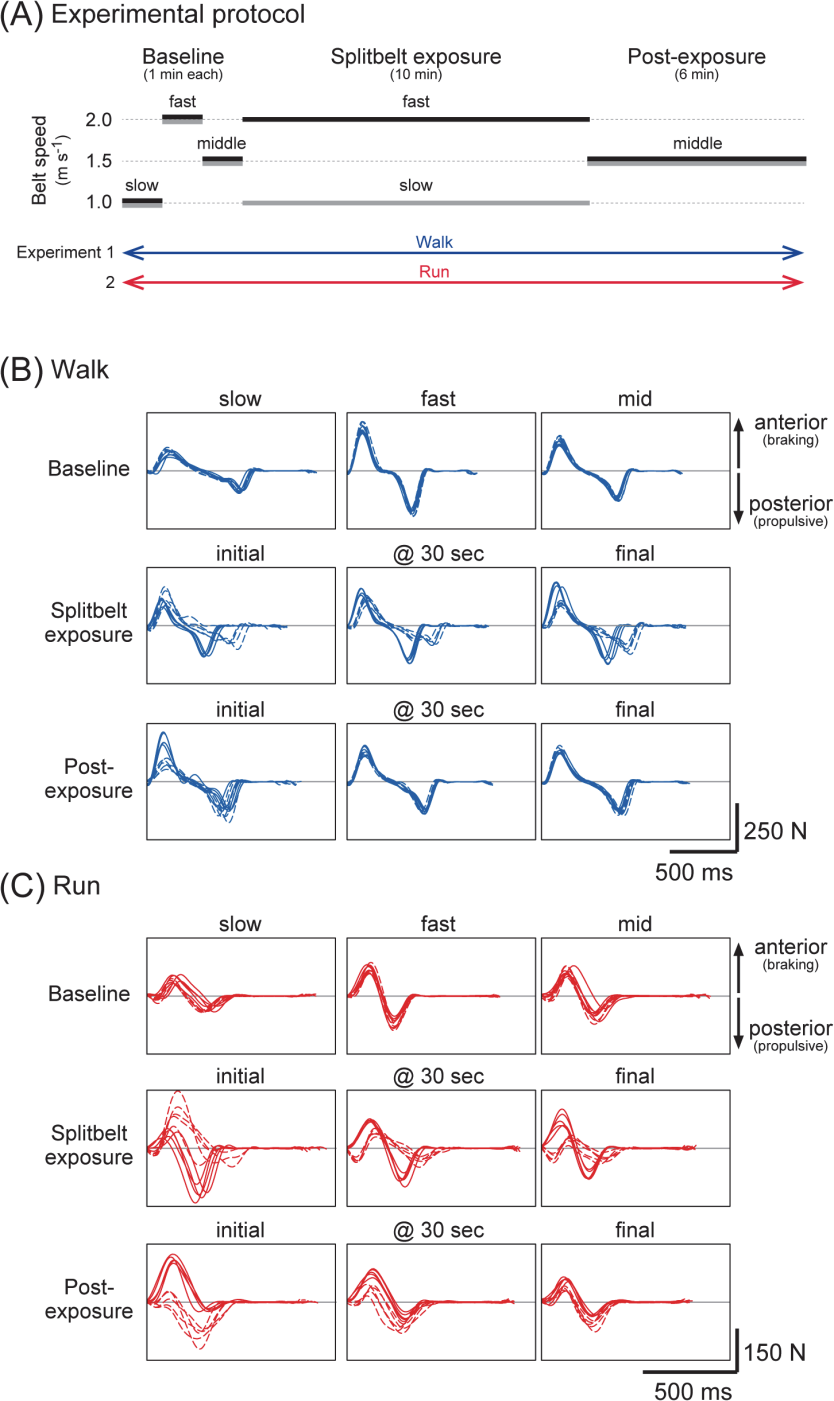
Experimental protocols and representative waveforms of the anterior-posterior ground reaction force (GRF). (A) In the experiments, subjects walk (experiment 1) or run (experiment 2) in the given speeds of treadmill. (B), (C) Each frame represents the GRF waveforms for five consecutive stride cycles at different time points in a single subject while walking (B) and running (C). The solid lines represent the fast side and the dashed lines are those of the slow side during the split-belt exposure.

Fig. 1(A) illustrates the experimental protocol. Each subject participated in two experiments, which were assigned in random order. Experiments 1 and 2 consisted of walking and running, respectively. Basically, subjects went through 10-minutes adaptation periods with the treadmill driven in split (split-belt exposure) and the behaviors in the respective gaits before and after the split-belt exposure were compared in tied. The velocities were determined on the basis of our preceding study [10], in which subjects could both walk and run comfortably. During the baseline, the velocities were 1.0 (slow), 2.0 (fast), and 1.5 m s^−1^ (middle) (1 minute each in duration). During the split-belt exposure, velocity of one belt (left) was adjusted to 2.0 m s^−1^ and the other (right) to 1.0 m s^−1^ (therefore, 2:1 in ratio). Subjects walked or ran under the novel split-belt physical constraint for 10 minutes. Following the 10-minute split-belt exposure, the belts were returned to the tied mode (at 1.5 m s^−1^). This period persisted for 6 minutes. Between each period, the velocities were changed continuously (without stopping) with acceleration and deceleration of 0.5 m s^−2^. The subjects were informed beforehand of these changes. The subjects were instructed to walk or run normally and to refrain from looking down at the treadmill belts to avoid receiving any visual information about the velocity.

For safety, one experimenter stood by the treadmill during the experiment. The treadmill had handrails to minimize the risk if the subjects lost their balance, but all of the subjects were able to satisfactorily complete the experiments without using the handrails.

### Data recordings and analysis

Three orthogonal components (mediolateral (Fx), antero-posterior (Fy), and vertical (Fz)) of the ground reaction force (GRF) were measured using two force sensors mounted underneath each of the treadmill belts. The force data were low-pass filtered at 8 Hz and digitized at a sampling frequency of 1 kHz using an analog-to-digital converter (Power Lab; AD Instruments). A custom-written program (VEE pro 9.0, Agilent Technologies) was used to analyze the force data. Using the vertical Fz component, stride cycles were determined on the basis of the moments of foot contact and toe off. For each stride cycle, peak absolute values in the antero-posterior Fy component were calculated for both the anterior (braking) and the posterior (propulsive) components (see Fig. 1(B)-(C) for representative waveforms). Differences in the peak forces between the sides (hereafter, termed as the degree of asymmetry) were calculated for both the anterior and the posterior components. For comparison between walking and running, since the cadence (therefore, the stride cycles taken) is different between the gaits along with possible differences between subjects, the data were averaged over stride cycles in 10-second bins. The averaged data were then normalized relative to the respective mean value under the middle velocity in the baseline period. Since the gaits could be perturbed by the acceleration or deceleration that occurred when the velocity changed, data for the first stride cycle after the velocity changed was excluded from later analysis.

To address rates of adaptation, the number of stride cycles that were required to adapt and de-adapt were calculated. On an individual basis, the stride to stride values for the degree of asymmetry (for both anterior and posterior components) were smoothed by using a 5-point moving average; the number of stride cycles the subjects took to reach plateau were determined for each parameter. The plateau was defined as the mean ± SD for each parameter over the last 50 stride cycles of the respective epoch (adaptation or de-adaptation).

Statistical analyses were performed for the degree of asymmetry in the anterior and the posterior forces at different time points (average taken over the 10 sec window) during the post-exposure periods (0–10 sec, 10–20 sec, 20–30 sec, 1 min (50 sec-1 min), 1 min 30 sec (1 min 20 sec-1 min 30 sec), 2 min (1 min 50 sec-2 min), 4 min (3 min 50 sec-4 min), and 6 min (5 min 50 sec-6 min)). A two-way analysis of variance (ANOVA) with repeated measures (factors: gait (walk or run) and time points) was used to test for statistically significant differences. When the ANOVA gave significant results, Bonferroni’s post-hoc comparisons were performed to test for differences between gaits. For the number of stride cycles taken to adapt and de-adapt, a paired Student’s t test was used to compare the differences between walking and running for each parameter. Data are presented as the mean and standard error of the mean (mean ± SEM). Stride-by-stride data are presented as the mean and standard deviation (mean ± SD). Statistical differences were accepted as significant when P < 0.05.

## Results

Panels (B) and (C) of Fig. 1 portray representative waveforms of the antero-posterior (Fy) GRFs for five consecutive stride cycles at different time points for walking (B) and running(C). Although minor, there are slight side-to-side differences in the forces at the baseline (treadmill in tied mode) both in walking and running. With exposure to the split treadmill mode with different velocity between the limbs, prominent asymmetry in the waveforms emerge in both gaits. As the gaits continue in the physical constraint over minutes, the shapes of the waveforms undergo significant changes. Interestingly, at the end of the split-belt exposure periods, the shapes of the waveforms resemble those observed under the respective velocity during the baseline period under the tied belt mode. For example, the waveforms in the fast side (solid lines in the split-belt exposure-final period) are similar to those seen in the fast baseline period and those for the slow side in the slow baseline period.

Similar to our preceding study [10], all subjects reported their movement as being perturbed during the post-exposure periods (especially at the beginning) for both walking and running. Indeed, although the velocities of the two treadmill belts were identical to those present during the baseline (mid) period, the resultant GRF waveforms differed to a great extent from those of the baseline and exhibited a prominent asymmetry. The profile of the alterations in the waveforms afterward, however, differed between walking and running as well as in the two GRF components. At 30 sec post-exposure, in contrast to the waveforms present during running, in which both anterior and posterior components were highly asymmetrical, the posterior components present during walking already restored symmetry.

**Fig 2:**
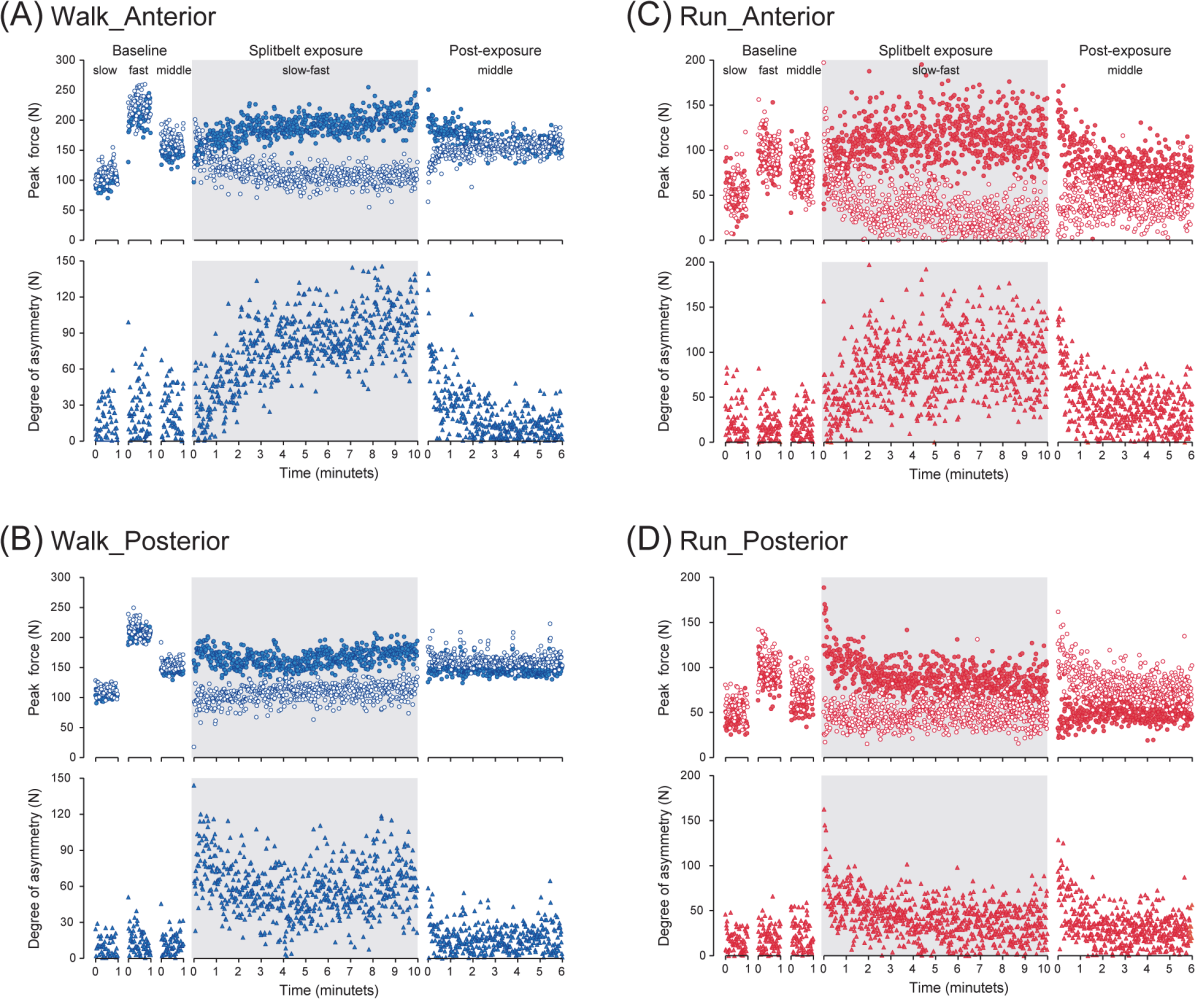
Stride-to-stride profile of the GRF. Graphs represent the anterior (A, C) and the posterior (B, D) components while walking (A, B) and running (C, D). Graphs in the upper rows are the peak values of both sides (filled circles: fast side and open circles: slow side under the split-belt exposure) and those in the lower rows are the differences in the peak force between the fast and the slow sides.

The overall trends described above in the GRF waveforms are more minutely plotted over the course of the experiments in Fig. 2, panels (A)-(D). Overall, alterations in the GRF differed between the anterior and the posterior components both during and after the split-belt exposure. Although different in magnitude, the profile of the anterior components showed similar trends for walking and running (Fig. 2, panels (A) and (C)). With exposure to the split-belt mode, the anterior components, which exhibited relatively equal values initially, developed clearly adaptive curves over minutes and differed from each other (therefore resulting in a major asymmetry between the sides). During the final phases of the split-belt exposure, the forces on each side came close to those seen around the baseline with the respective velocities (slow side relative to the slow baseline and fast side relative to the fast baseline). With return to the tied mode, the forces showed a significant asymmetry that subsequently returned to that seen during the baselines. This return required about 2 minutes and demonstrated a clear de-adaptive behavior.

Alterations in the posterior component, on the other hand, showed a different trend between walking and running (Fig. 2, panels (B) and (D)). Split-belt exposure resulted in prominent asymmetry in the initial peak posterior forces, both in walking and in running. The subsequent profile however, showed an adaptive aspect only in running. The walking profile, rather demonstrated a more linear shift over the 10 minute split-belt exposure period. When the velocities were returned to the tied mode for the post-exposure periods, only running exhibited a definite aftereffect, followed by gradual de-adaptive behavior. In contrast, walking showed only minor alterations in contrast to those seen in running during the baseline period with an identical velocity.

**Fig 3:**
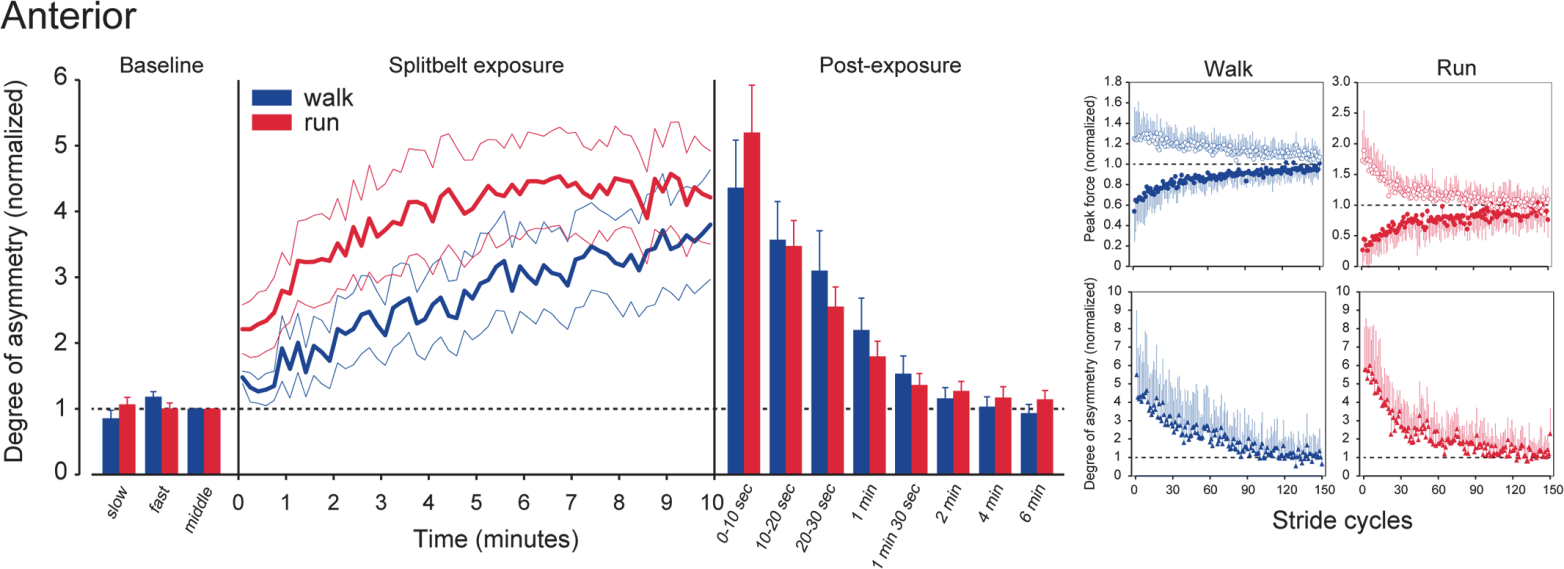
Comparison of the anterior component of the GRF between walking and running. (Left panel) Degree of asymmetry in the peak anterior force at different time points. Data are normalized to those during the baseline. Grey graphs represent the data for walking and black graphs are those for running. Error bars (thin lines during the split-belt exposure) represent the standard error of the mean. (Right panels) Mean stride-to-stride changes in the anterior and the posterior components of the GRF. Data are for the first 150 stride cycles of the post-exposure periods for walking (left columns) and running (right columns). Graphs in the upper rows of each panels represent the peak values of both sides (filled circles: “fast” side and open circles: “slow” side—under the split-belt exposure). Those in the lower rows are the differences in the peak force between the fast and the slow sides. Error bars indicate standard deviations.

**Fig 4:**
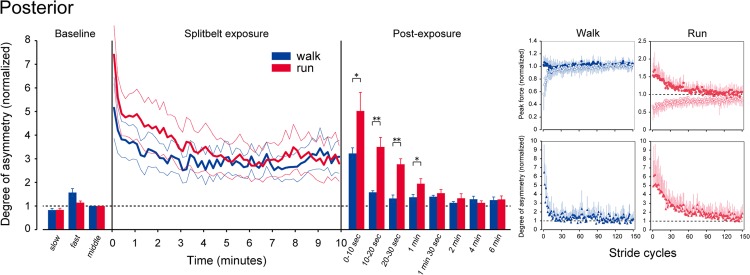
Comparison of the posterior component of the GRF between walking and running. (Left panel) Degree of asymmetry in the peak posterior force at different time points. Data are normalized to those during the baseline. Grey graphs represent the data for walking and black graphs are those for running. Error bars (thin lines during the split-belt exposure) represent the standard error of the mean. Asterisks indicate significant differences between walking and running (* P < 0.05, ** P <0.01). (Right panels) Mean stride-to-stride changes in the anterior and the posterior components of the GRF. Data are for the first 150 stride cycles of the post-exposure periods for walking (left columns) and running (right columns). Graphs in the upper rows of each panel represent the peak values of both sides (filled circles: “fast” side and open circles: “slow” side—under the split-belt exposure). Those in the lower rows are the differences in the peak force between the fast and the slow sides. Error bars indicate standard deviations.

The trends shown above are representative of those seen in the mean data. Figs. 3 and 4 depict comparisons of mean values in the degree of asymmetry between walking and running at different time points (left panels). Among the various differences, most notable is the emergence of an aftereffect during the first few minutes of the post-exposure periods. In contrast to the anterior component, where there were prominent aftereffects in both walking and running (statistically significant result for time point: F(7,56) = 30.536, p<0.001), in the posterior component the magnitude of the aftereffect was quite different between the gaits. While running shows a clear aftereffect, the degree of asymmetry in walking remains quite low except for the first 10 second period. An ANOVA comparison gave significant results for the posterior component (gait: F(1,8) = 11.557, p<0.01; time point: F(7,56) = 35.996, p<0.001, gait×time point: F(7,56) = 7.946, p<0.001). The degree of asymmetry was significantly greater during running for up to 1 minute.

**Fig 5:**
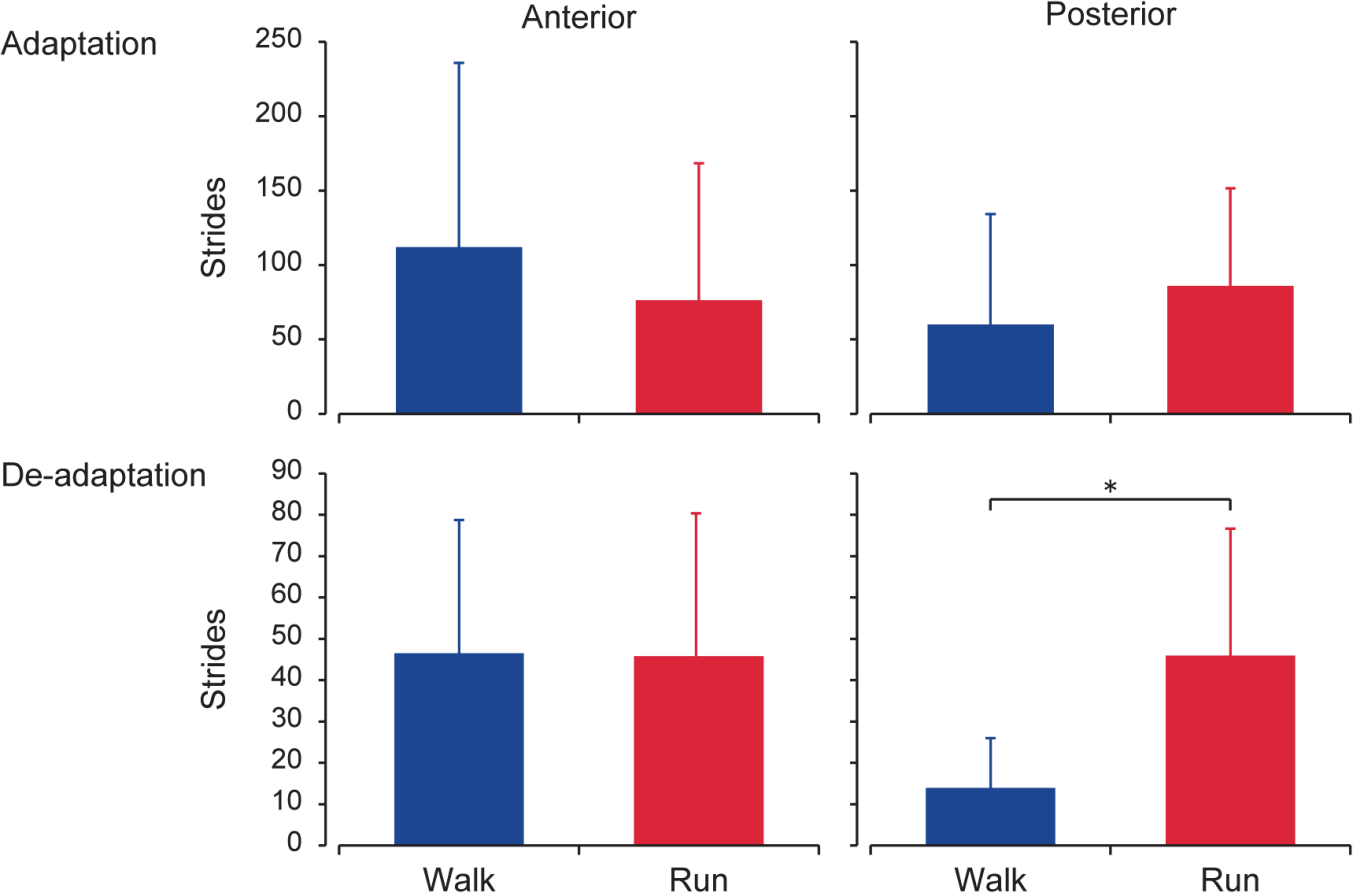
Comparison in the number of stride cycles taken to adapt and to de-adapt. Data are counted by the anterior braking (left panels) and the posterior propulsive (right panels) component of the GRF for both walking (blue bars) and running (red bars). Error bars indicate standard deviation. An asterisk indicates a significant difference between walking and running (* P < 0.05).

To precisely track the emergence of the aftereffect, mean values of the peak forces (side-to-side) and the degree of asymmetry are plotted on a stride-by-stride basis for the first 150 stride cycles of the post exposure periods in the right panels of Figs. 3 and 4. Here again, the anterior component shows obvious de-adaptive behavior, both in walking and running. For the posterior component, however, only running demonstrated such a profile. For walking, prominent asymmetry was evident for about 10 stride cycles; no further asymmetries were observed over the course of the experiment. These trends are well reflected in the number of stride cycles taken to adapt and de-adapt (Fig. 5). With the relatively similar numbers seen between walking and running both in the anterior component and in the posterior component of adaptation (upper panels) as well as in the anterior component of de-adaptation (left lower panel), the posterior component of de-adaptation demonstrates a significant difference between the gaits (P < 0.05).

## Discussion

The purpose of this study was to investigate the characteristics of split-belt locomotor adaptations in human running by focusing on the anterior braking and posterior propulsive components of the GRFs. Adaptation and de-adaptation in running were compared with those in walking. In accordance with our preceding results, robust adaptive and de-adaptive aspects were evident in the anterior braking force while the posterior propulsive component showed only short-term aftereffect (lasting less than 10 seconds) accompanied by largely reactive responses in walking. For running, in distinction with the results from walking, adaptive and longer lasting (up to around 1 minute) de-adaptive aspects were emergent in both anterior braking and posterior propulsive components. These results therefore demonstrate a possible difference in motor strategies underlying split-belt locomotor adaptation in the two main forms of human locomotion.

### Locomotor adaptation and the underlying neural mechanisms

In locomotion, humans and other animals can transiently adjust their movement patterns to meet changing task or environmental demands. Moreover, repetition of the movement tasks or those under constant environmental constraints over minutes results in more persisting adjustments in motor output and hence an emergence of movement error upon return to normal condition without constraints (aftereffect) [12,18,19]. Such aspects have been investigated using particular kinds of physical constraint such as a curved path [20], a robotically- induced resistance [19], an elastic force field [18], and split-belt treadmills [12,21]. The emergence of these phenomena can be explained by the formation of a cerebellar “internal model”. This model involves the recalibration of the motor output on the basis of trial-and-error processes which occurs via a comparison between the expected and the resultant movements [1,22]. Indeed, the importance of cerebellar function on locomotor adaptation has been demonstrated both in animal [23] and human studies [8]. Long lasting motor adaptability upon walking on split-belt treadmill is impaired in both cats [23] and humans [8] with cerebellar deficits. In healthy subjects, Jarayam and colleagues [5], using a transcranial magnetic stimulation (TMS), demonstrated an involvement of cerebellar function in split-belt treadmill locomotor adaptations for human walking. They particularly revealed that the cerebellar involvement was specific to walking on the split-belt. This cerebellar involvement could not be simply attributed to the task difficulty resulting from the changing velocities of the treadmill.

### Distribution of reactive feedback and adaptive feedforward strategies in locomotor adaptation

Even more importantly, the emergence of the adaptive and de-adaptive behaviors is specific to the phases of locomotion. It has been proposed that both reactive feedback and adaptive feedforward strategies are used depending on gait phases where the timing is dependent on the modality of the given physical constraint [18,19]. Locomotor adaptation in split-belt treadmill walking also exhibits a distribution of both control strategies throughout the stride cycle. Based on measurement of the GRF while walking on a split-belt treadmill, Ogawa et al. [11] revealed that feedforward control were merged to the earlier part of the stance phase (around heel contact), while feedback strategies occurred during the latter phase of the stance. They also noted that in contrast to the anterior braking component that exhibited slow adaptive and de-adaptive changes upon changing velocities, the posterior propulsive component showed only a fast reactive modification during walking [11]. To note, the velocities used in the study [11] were 0.5 and 1.0 m s^−2^ for “tied” condition, and 0.5–1.0 m s^−2^ for “split”, therefore different from those used in the present study. Another group has addressed gait adaptations by focusing predominantly on the temporal and spatial aspects of locomotion [12]. In a similar gait adaptation task, certain parameters such as step time, double support time, and the relative timing of two leg movement showed adaptive behavior and a subsequent aftereffect, while others, including modified stride length (anterior-posterior distance traveled by the foot during the stance phase) and stance time were reactive with only transient modifications [12]. With the possible variation in gait strategies (that is, relative contribution of both temporal and spatial adjustments to a given gait speed) both within and among subjects, use of these parameters may potentially underestimate the emergence of the adaptive phenomena. We have therefore chosen to focus on the analysis of the ground reaction forces [10,11]. However, this approach does create the potential limitation of not being able to address the swing-related aspects of locomotor adaptation.

Given the results of the preceding studies, though speculative, the difference in the adaptive and de-adaptive behaviors between walking and running may reflect different strategies by the CNS in terms of the feedback and feedforward control. Despite the similarity with well-coordinated joint movements both within and between the two multisegmental limbs, what functional differences exist between the two gaits? Possible differences in motor strategies underlying this adaptation are discussed below, based on known neural mechanisms of walking and running.

### Neural mechanisms underlying walking and running

In the literature, locomotive movements are understood to be generated by specialized neural mechanisms in the CNS (for a review, see [24]). Moreover, recent studies have demonstrated independence of the neural mechanisms underlying different locomotive modes. Both direct recordings of neural activity in larval zebrafish [25] and experimental lesions of specific groups of neurons in mice [26] reveal that different groups of spinal interneurons underlie locomotion that occurs with different movement frequencies. In the supraspinal mechanisms, it has been demonstrated that stimulation to the mesencephalic locomotor region results in the emergence of different gait modes (walk, trot and gallop in cats; stepping and swimming in salamander) depending on the stimulation frequency [27–29].

In humans, a study of motor adaptation revealed that walking and running are controlled by distinct functional units being capable of each mode rather than sharing a common neural mechanism [10]. Regarding the basic motor patterns of walking and running, a number of studies have provided clear descriptions on the basis of muscle use [16,17,30,31]. The studies of Cappellini et al. [16] and Ivanenko et al. [17] analyzed activation patterns of 32 muscles during walking and running over a range of speeds. These studies revealed that the timing of activation in one out of five basic components distributed differently through the movement cycles depending on the gait mode and irrespective of the speed. Particularly, since the timing shift occurred within stance phase, this may account for one possible explanation for the present results in which the GRF component showed a different profile between the two gaits.

Kinetically, walking and running during the stance phases are characterized as “inverted” pendulum and “bouncing gait” [32]. In the inverted pendulum gait (walking), the center of mass (COM) is assumed to vault over a stiff leg. The bouncing gait (running), on the other hand, utilizes an elastic spring-like behavior of the limb. Walking and running therefore, use different forms of energy transformation, especially for horizontal braking upon foot contact and for propulsion at toe off. Through the stance phases, the amplitude of spinally-mediated monosynaptic H-reflex of the soleus muscle is known to be more suppressed in running than in walking, despite a greater level of background muscle activity in running [33]. These results indicate a greater contribution of peripherally-mediated reactive control in walking as compared with running. The soleus muscle has been shown to be an important contributor to forward propulsion in both walking [34] and running [35]. This raises the possibility that there is an increased contribution of peripherally-mediated reactive control in walking and thus a relatively greater contribution of the higher centers in running. It is possible that these differences in the neural strategies between the two gaits may have accounted for the emergence of the different behavior in the propulsive component of the GRF.

To summarize, with the similarity in the adaptation and de-adaptation of the anterior braking component, walking and running showed prominent difference in the posterior propulsive GRF component. In running, therefore, the factors contributing to the production of the posterior GRF component may additionally account for a key element for acquisition of motor patterns. This knowledge provides basic information to further studies, such as those focusing on task- or context- specificity of human gait and those aiming at performance enhancement in athletic training and/or in gait rehabilitation after particular motor dysfunction.
